# HIV-1 Tat Protein Enters Dysfunctional Endothelial Cells via Integrins and Renders Them Permissive to Virus Replication

**DOI:** 10.3390/ijms22010317

**Published:** 2020-12-30

**Authors:** Aurelio Cafaro, Giovanni Barillari, Sonia Moretti, Clelia Palladino, Antonella Tripiciano, Mario Falchi, Orietta Picconi, Maria Rosaria Pavone Cossut, Massimo Campagna, Angela Arancio, Cecilia Sgadari, Claudia Andreini, Lucia Banci, Paolo Monini, Barbara Ensoli

**Affiliations:** 1 National HIV/AIDS Research Center, Istituto Superiore di Sanità, V.le Regina Elena 299, 00161 Rome, Italy; aurelio.cafaro@iss.it (A.C.); sonia.moretti@iss.it (S.M.); clelia.palladino@iss.it (C.P.); antonella.tripiciano@iss.it (A.T.); mario.falchi@iss.it (M.F.); orietta.picconi@iss.it (O.P.); mariarosaria.pavonecossut@iss.it (M.R.P.C.); massimo.campagna@iss.it (M.C.); angela.arancio@iss.it (A.A.); cecilia.sgadari@iss.it (C.S.); paolo.monini@iss.it (P.M.); 2 Department of Clinical Sciences and Translational Medicine, University “Tor Vergata”, 00161 Rome, Italy; barillar@uniroma2.it; 3 CERM (Magnetic Resonance Center), University of Florence, 50019 Florence, Italy; andreini@cerm.unifi.it (C.A.); banci@cerm.unifi.it (L.B.)

**Keywords:** integrins, endothelial cells, inflammatory cytokines, HIV-1 Tat protein, cellular uptake, HIV-1 target cells

## Abstract

Previous work has shown that the Tat protein of Human Immunodeficiency Virus (HIV)-1 is released by acutely infected cells in a biologically active form and enters dendritic cells upon the binding of its arginine-glycine-aspartic acid (RGD) domain to the α5β1, αvβ3, and αvβ5 integrins. The up-regulation/activation of these integrins occurs in endothelial cells exposed to inflammatory cytokines that are increased in HIV-infected individuals, leading to endothelial cell dysfunction. Here, we show that inflammatory cytokine-activated endothelial cells selectively bind and rapidly take up nano-micromolar concentrations of Tat, as determined by flow cytometry. Protein oxidation and low temperatures reduce Tat entry, suggesting a conformation- and energy-dependent process. Consistently, Tat entry is competed out by RGD-Tat peptides or integrin natural ligands, and it is blocked by anti-α5β1, -αvβ3, and -αvβ5 antibodies. Moreover, modelling–docking calculations identify a low-energy Tat-αvβ3 integrin complex in which Tat makes contacts with both the αv and β3 chains. It is noteworthy that internalized Tat induces HIV replication in inflammatory cytokine-treated, but not untreated, endothelial cells. Thus, endothelial cell dysfunction driven by inflammatory cytokines renders the vascular system a target of Tat, which makes endothelial cells permissive to HIV replication, adding a further layer of complexity to functionally cure and/or eradicate HIV infection.

## 1. Introduction

By mediating the adhesive interactions occurring between endothelial cells and the vessel basement membrane, integrins exert important roles in either the maintenance of vascular integrity or the formation of new vessels [1,2]. Accordingly, alterations in integrin expression and/or function occur in endothelial cell dysfunction and associated morbidities [1,3,4,5,6,7].

Among the inducers of endothelial cell dysfunction are inflammatory mediators including interleukin (IL)-1β, tumor necrosis factor (TNF)-α, and interferon (IFN)-γ; of importance, these cytokines can alter the expression and/or function of integrins, such as α5β1 or αvβ3, which are key to endothelial cells [8,9,10,11,12].

In this regard, HIV-infected individuals display a chronic systemic inflammation and high IL-1β, TNF-α or IFN -γ plasma levels, even when they are treated with the combination antiretroviral therapy (cART) which reduces Human Immunodeficiency Virus (HIV)-1 replication or infectivity [13,14,15,16]. Indeed, in HIV infection, endothelial cells are chronically activated, release vascular adhesion molecules and angiogenic factors, and undergo numerous additional changes characteristic of endothelial dysfunction, which, in turn, has been proposed to contribute to the increased incidence of cardiovascular diseases observed in the pre-ART and in the ART era, respectively [17,18,19,20]. Moreover, although mainly associated to cardiovascular diseases, endothelial dysfunction occurs in all the AIDS and non-AIDS-associated morbidities [21]. In fact, abnormal angiogenesis and altered permeability of the blood–brain barrier associated to central and peripheral nervous system vasculopathies occur at high frequency in HIV infected individuals and contribute to the onset of tumors and dementia [22].

As for inflammatory cytokines, several HIV proteins continue to be expressed even during effective cART [23,24,25,26,27], which may contribute to endothelial dysfunction and increased cardiovascular disease risk [18,19,20,21,22,23,24,25,26,27,28]. Notably, the development of cardiovascular disease has been reported in mouse and rat models transgenic for HIV genes in the absence of virus replication [29,30], underscoring the role of HIV proteins per se.

Of them, HIV-1 Tat, the earliest protein to be produced upon virus entry and even prior to virus integration, has been shown to severely affect endothelial cell functionality [31,32]. Although fundamental for HIV gene transcription, the majority (about 65%) of the Tat protein synthesized during acute infection is released by the infected cells in the extracellular milieu in a biologically active form [33,34,35,36]. This occurs through exocytosis, via a leaderless secretory pathway, in the absence of cell death or cell permeability changes [33,34].

Notably, nano-micromolar concentrations of extracellular Tat protein, which are comparable to those detected in vivo in tissues, selectively enter dendritic cells upon the engagement of the α5β1, αvβ3, and αvβ5 integrins [37]. In doing so, Tat drives dendritic cell maturation toward a Th-1 polarizing phenotype, further modulating T cell responses [38]. This phenomenon greatly differs from the ability of micromolar concentrations of Tat to enter virtually all cell types via the binding of Tat basic residues to the heparan-sulfate proteoglycans (HSPG) of the cell membrane [39].

In addition to dendritic cells, Tat influences also endothelial cells, and this occurs through a variety of modes [31,32]. As first, it is noteworthy that the extracellular Tat protein can promote the locomotion, adhesion, and growth of endothelial cells after their activation with TNF-α, IL-1β, and IFN-γ, which increase the expression or activity of the arginine-glycine-aspartic acid (RGD)-binding α5β1 and αvβ3 integrins [40,41,42]. In doing so, Tat increases the frequency and expressiveness of Kaposi’s sarcoma (KS), an angioproliferative tumor frequent in HIV-infected individuals [41,43,44]. The finding that Tat, αvβ3, and α5β1 are highly expressed in AIDS-associated KS lesions [39] suggests that these mechanisms are indeed operating in vivo.

In this context, it is worth noticing that Tat itself can directly induce the expression of inflammatory cytokines [45,46,47,48,49]. Tat also increases leukocyte adhesiveness to endothelial cells and trans-endothelial migration, as it induces the expression of intercellular adhesion molecule-1 (ICAM-1), vascular adhesion molecule-1, and E-selectin on the endothelial cell surface [50]. In addition to favoring the extravascular dissemination of HIV-1, the newly synthesized adhesion molecules place HIV-infected leukocytes in close proximity with endothelial cells.

Based on this evidence, here, we have evaluated whether extracellular Tat protein could enter endothelial cells via the α5β1, αvβ3, or αvβ5 integrins, and whether this could support HIV replication in these otherwise poorly susceptible cells.

## 2. Results

### 2.1. Biologically Active Tat Enters Primary Endothelial Cells in a Dose-, Time-, and Activation-Dependent Fashion

To determine whether the Tat protein of HIV-1 could enter quiescent and/or activated endothelial cells, primary human umbilical vein endothelial cells (HUVEC) were cultured in the presence or absence of human recombinant IL-1β, TNF-*α*, and IFN-*γ*, which were combined at doses as found in inflammatory microenvironments. Endothelial cell activation was confirmed throughout the course of the whole study by evaluating the cell surface expression of ICAM-1 (CD54), which was >90% (data not shown).

HUVEC, both inflammatory cytokine (IC)-activated (IC-HUVEC) and non-activated (HUVEC), were incubated in suspension with biologically active Tat protein at concentrations compatible with those detected in specimens from HIV-infected individuals [51,52,53,54]. Then, the intracellular Tat content was evaluated by staining with anti-Tat antibodies (Ab) and flow cytometry.

**Figure 1:**
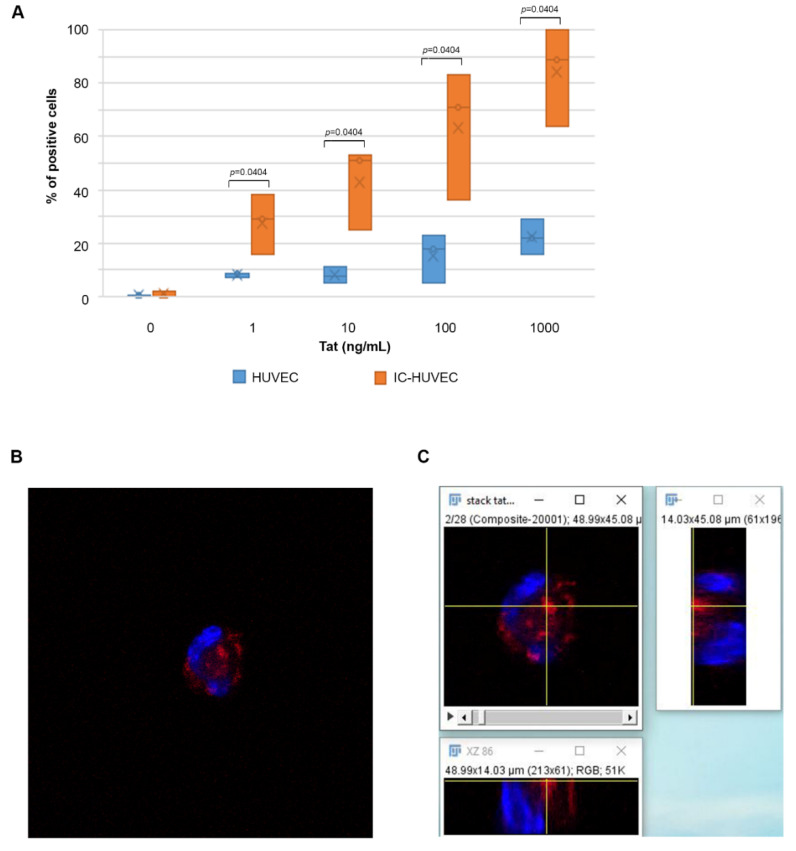
Extracellular Tat protein is efficiently taken up by activated endothelial cells. In (**A**) human umbilical vein endothelial cells (HUVEC, blue plots) or inflammatory cytokine (IC)-activated (IC-HUVEC (orange plots) were incubated for 10 min in medium containing serial concentrations (1–1000 ng/mL) of biologically active Tat or its suspension buffer (PBS-0.1% BSA). Intracellular Tat content was evaluated by flow cytometry after staining with affinity-purified rabbit anti-Tat polyclonal Ab (or isotype control), as described in Section 4 Materials and Methods. Non-permeabilized cells were employed as an additional control. Results are expressed as the percentage of positive cells, as compared to isotype-stained samples. Box-plot of data obtained from three independent experiments and analyzed by the Mann–Whitney nonparametric test are shown. Dots indicate individual measures. In (**B**,**C**), IC-HUVEC were incubated with rhodamine-labeled Tat (100 ng/mL) and treated as described in Section 4 Materials and Methods. Images are optical sections (optical thickness = 0.40 μm) collected from a confocal microscope showing cellular internalization of Tat (red). Blue indicates nuclei stained with DAPI. Scale bar = 15 μm.

Tat entered IC-HUVEC very rapidly and in a dose-dependent fashion. Specifically, when IC-HUVEC were exposed for 10 min to 1, 10, 100, or 1000 ng/mL of Tat, the percentage of positive cells was 29, 51, 71, and 89%, respectively (Figure 1A). In sharp contrast, the proportion of untreated HUVEC taking up Tat was virtually absent at low (nanomolar) Tat concentrations (1–10 ng/mL), whereas only 22% of HUVEC stained positive at Tat 1000 ng/mL (Figure 1A). Thus, IC-HUVEC internalized the Tat protein much more efficiently than HUVEC at all Tat concentrations tested (*p* = 0.0404). Based on these results, IC-HUVEC were chosen for the subsequent experiments.

Then, time course experiments were performed to determine the kinetics of Tat entry. Of interest, irrespective of the protein concentration, Tat uptake by IC-HUVEC was already maximal after the shortest time (5 min) of exposure to the protein, and it started to slowly decline after 30 min, except for the highest Tat concentration (Figure S1). In this regard, virtually all cells (>95%) stained positive for intracellular Tat upon 5 min exposure to 1000 ng/mL of the protein, and no changes were recorded at the subsequent time points (Figure S1), suggesting the involvement of different receptor(s) and/or pathway(s) of internalization and processing of the protein, as previously reported to occur for monocyte-derived dendritic cells [37].

Anti-Tat Abs did not stain non-permeabilized cells incubated with Tat (data not shown), indicating that (i) cell surface-bound Tat was totally removed by treating cells with trypsin; (ii) most protein was internalized; and (iii) neither the trypsin used to remove cell surface-bound Tat nor the non-enzymatic procedure utilized to suspend IC-HUVEC altered the cell membrane permeability.

However, to rule out the interference of cell detachment or trypsinization in the Tat uptake process, adherent IC-HUVEC were exposed to rhodamine-labeled Tat. Then, entry of the protein was visualized by confocal microscopy, as described in Section 4 Materials and Methods. As shown in Figure 1B, the orthogonal view revealed the presence of Tat in an introflexion of the nucleus, which is a finding supported by the tomographic analysis of the confocal image (Figure S2). Taken together, the data indicate that biologically active Tat enters both suspended and adherent cells.

### 2.2. Tat Entry in IC-HUVEC Is Reduced by Low Temperature or Tat Oxidation

**Figure 2:**
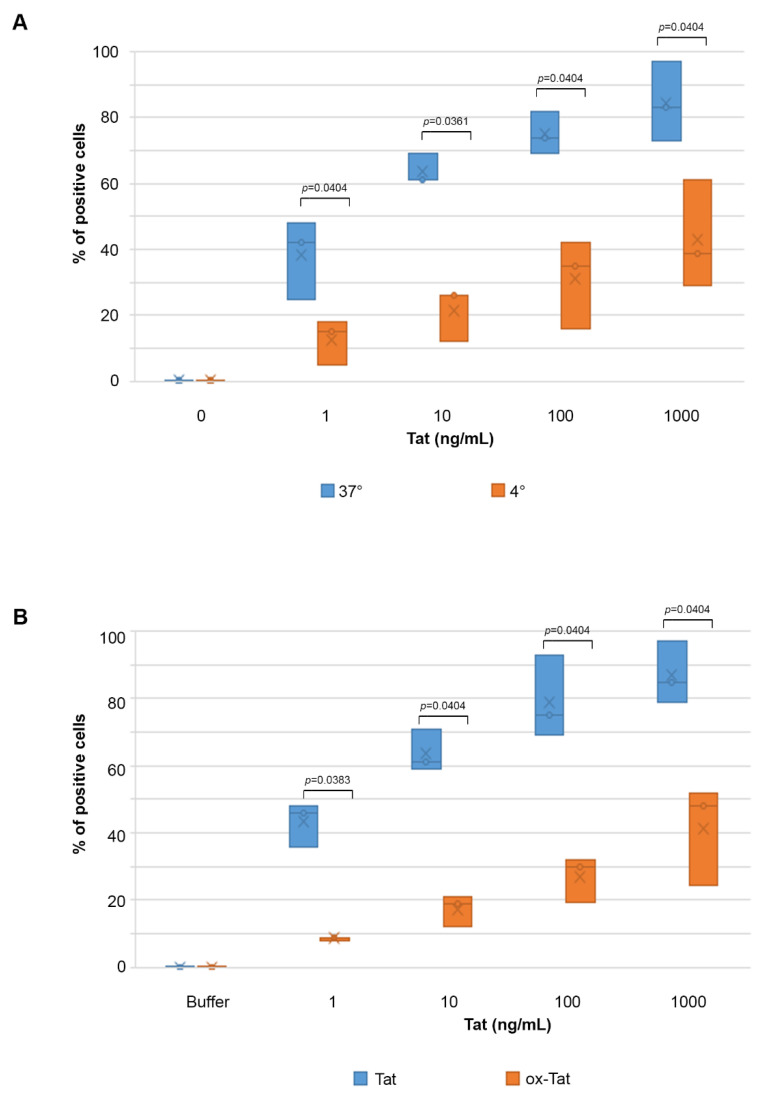
Entry of Tat in IC-HUVEC is reduced by low temperatures or Tat oxidation. (**A**) IC-activated HUVEC were incubated for 10 min at 37 °C (blue plots) or 4 °C (orange plots) with biologically active Tat (1–1000 ng/mL) or its buffer (0 ng/mL). (**B**) IC-HUVEC were incubated for 10 min with 1–1000 ng/mL of biologically active (blue plots) or inactive, oxidized (orange plots) Tat. For both (**A**,**B**), the detection of intracellular Tat was performed as described in the legend to Figure 1, and the results are expressed as the percentage of positive cells. Box-plot of data obtained from three independent experiments and analyzed by the Mann–Whitney test are shown. Dots indicate individual measures. In all experiments, the fluorescence of permeabilized cells stained with anti-Tat Ab was compared to that of isotype-stained samples and non-permeabilized cells.

To further characterize the entry of Tat in IC-HUVEC, cells were exposed to different temperatures. In fact, previous work indicated that Tat uptake by human tumor cell lines or dendritic cells is strongly reduced or lost at low temperatures, or upon Tat oxidation and inactivation [37,55]. In agreement with these findings, the entry of Tat into IC-HUVEC was strongly reduced at 4 °C as compared to 37 °C. In particular, the entry of 1 or 10 ng/mL Tat into IC-HUVEC was inhibited by 64 and 57%, respectively, while cellular uptake of 100 or 1000 ng/mL Tat was reduced by 53% (*p* = 0.0404, Figure 2A).

Similarly, a marked reduction of Tat entry was observed upon Tat protein inactivation by oxidation (see Section 4 Materials and Methods). In fact, the entry of 1, 10, 100, or 1000 ng/mL of oxidized Tat into IC-HUVEC was reduced by 80, 69, 60, and 44%, respectively, as compared to native, biologically active Tat (*p* < 0.05, Figure 2B).

Taken together, these results indicate that an efficient uptake of Tat by IC-HUVEC requires a biologically active protein and it is likely to occur through receptor-mediated endocytosis, which is an energy-dependent process that is reduced at low temperatures, although additional pathway(s) may exist for higher Tat concentrations.

### 2.3. Uptake of Tat by Activated HUVEC Requires Both the RGD Domain and the Basic Region of Tat, and It Is Competed by Fibronectin and Vitronectin

**Figure 3:**
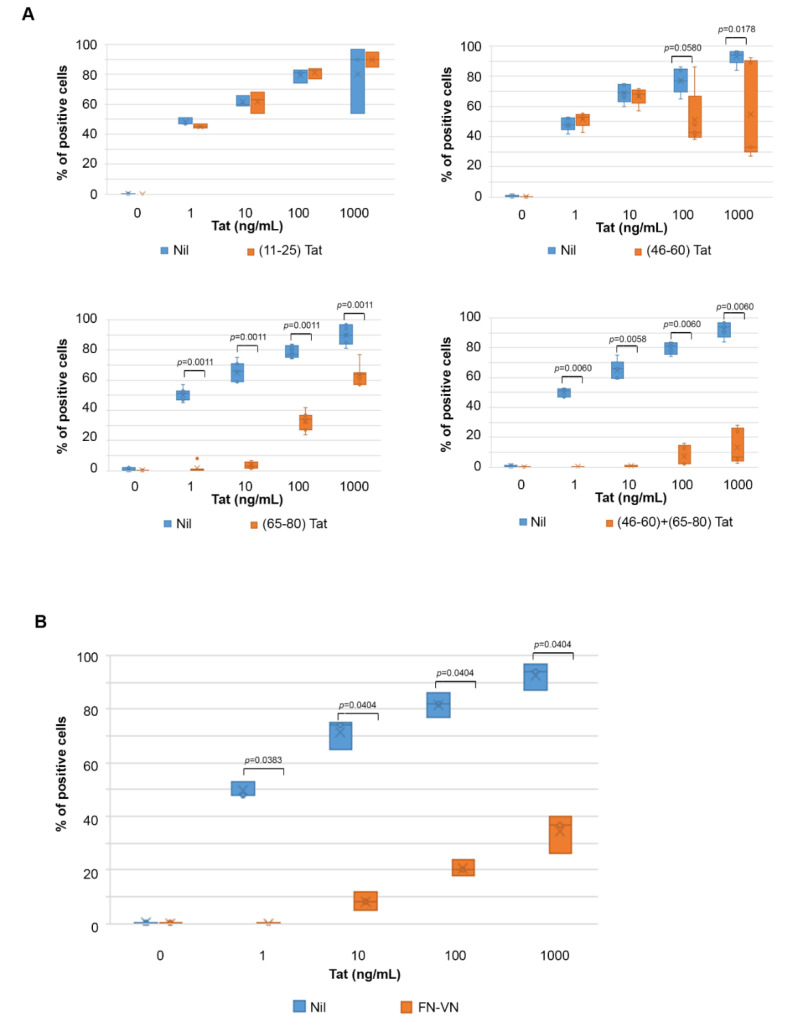
Role of Tat arginine-glycine-aspartic acid (RGD) and basic region in entry of Tat in IC-HUVEC, and entry blockade by fibronectin and vitronectin. In (**A**), IC-HUVEC were incubated for 2 h with 100 μg/mL of the (11–25) Tat-ACIDIC (upper left panel), (46–60) Tat-BASIC (upper right panel), (66–80) Tat-RGD (lower left panel) or combined (46–60) Tat and (66–80) Tat (lower right panel) peptides. Cells incubated with the peptide suspension buffer (PBS-0.1% BSA) were employed as controls. In (**B**), IC-HUVEC were incubated for 2 h with fibronectin (FN) and vitronectin (VN), combined at 50 μg/mL each. FN and VN dilution buffer (PBS-0.1% BSA) served as a negative control (Nil). For both (**A**,**B**), after pre-incubation, IC-HUVEC were exposed for 10 min to biologically active Tat (1–1000 ng/mL) or its suspension buffer (Nil). Intracellular Tat content was assessed by intracellular staining and flow cytometry as described in the legend to Figure 1. Results are expressed as the percentage of positive cells as compared to isotype-stained samples and non-permeabilized cells. Box-plot data obtained from three to six independent experiments and analyzed by the Mann–Whitney test are shown. Dots in box-plots indicate individual measures. Orange plots refer to IC-HUVEC incubated with Tat peptides (**A**) or combined FN and VN (**B**); blue plots refer to control IC-HUVEC.

Then, blocking experiments were performed to identify the Tat domains involved in the uptake of Tat by IC-HUVEC. Specifically, IC-HUVEC were incubated with a molar excess of Tat or Tat peptides spanning either the Tat RGD domain (Tat-RGD aa 66-80) or the Tat basic region (Tat-BASIC, aa 46-60). The peptide spanning the acidic domain of Tat (Tat-ACIDIC, aa 11-25) was used as control, since this Tat region does not bind the endothelial cell membrane [39,40,41,56,57]. Incubation with competitor peptides was followed by the addition of Tat at concentrations ranging from 1 to 1000 ng/mL. As shown in Figure 3A, the pre-incubation of IC-HUVEC with Tat-ACIDIC did not affect cellular uptake of any of the Tat concentrations tested. In contrast, prior incubation with Tat-RGD abolished IC-HUVEC uptake of 1 and 10 ng/mL of Tat, while the entry of 100 and 1000 ng/mL Tat was reduced by 56 and 29%, respectively (*p* = 0.0011 and *p* = 0.0010, Figure 3A). At variance with the Tat-RGD peptide, the Tat-BASIC peptide had no effect on the uptake of 1 and 10 ng/mL of Tat, whereas it reduced the uptake of 100 and 1000 ng/mL Tat by 44 and 65%, respectively (*p* = 0.0580 and *p* = 0.0178, Figure 3A). Consistent with these findings, the prior incubation of IC-HUVEC with combined Tat-BASIC and Tat-RGD peptides abolished the entry of Tat 1, 10, and 100 ng/mL, and it strongly reduced (−93%) the uptake of 1000 ng/mL of Tat (*p* = 0.0060, Figure 3A).

To verify whether Tat entry in IC-HUVEC is mediated by RGD-binding integrins, fibronectin and vitronectin, the natural ligands of these receptors, were used as competitors. Pre-incubation with fibronectin and vitronectin abolished IC-HUVEC uptake of 1 ng/mL Tat and reduced the uptake of 10, 100, and 1000 ng/mL Tat by 89, 76, and 61%, respectively (*p* = 0.0404, Figure 3B). This indicated that RGD-binding integrins are required for Tat entry and confirmed that at nano-micromolar concentrations, Tat entry is strictly dependent on the RGD domain of Tat.

Thus, the entry of low doses of Tat appears to be mediated exclusively by the Tat RGD region, whereas both the RGD and the basic region are involved in the entry of high concentrations of Tat.

### 2.4. Tat Entry in IC-HUVEC Is Blocked by Monoclonal Antibodies against the α5β1, αvβ3, and αvβ5 Integrins

Following the identification of the Tat domains that mediate the uptake of the protein by IC-HUVEC, intracellular staining and flow cytometry were employed to assess whether Tat entry into IC-HUVEC could be inhibited by monoclonal antibodies (mAbs) directed against the RGD-binding integrins α5β1 or αvβ3. MAbs were employed at a concentration (5 µg/mL) that abolishes IC-HUVEC adhesion onto immobilized fibronectin or vitronectin (data not shown). A mAb directed against the endothelial cell marker CD31 was employed as a control of specificity.

**Figure 4:**
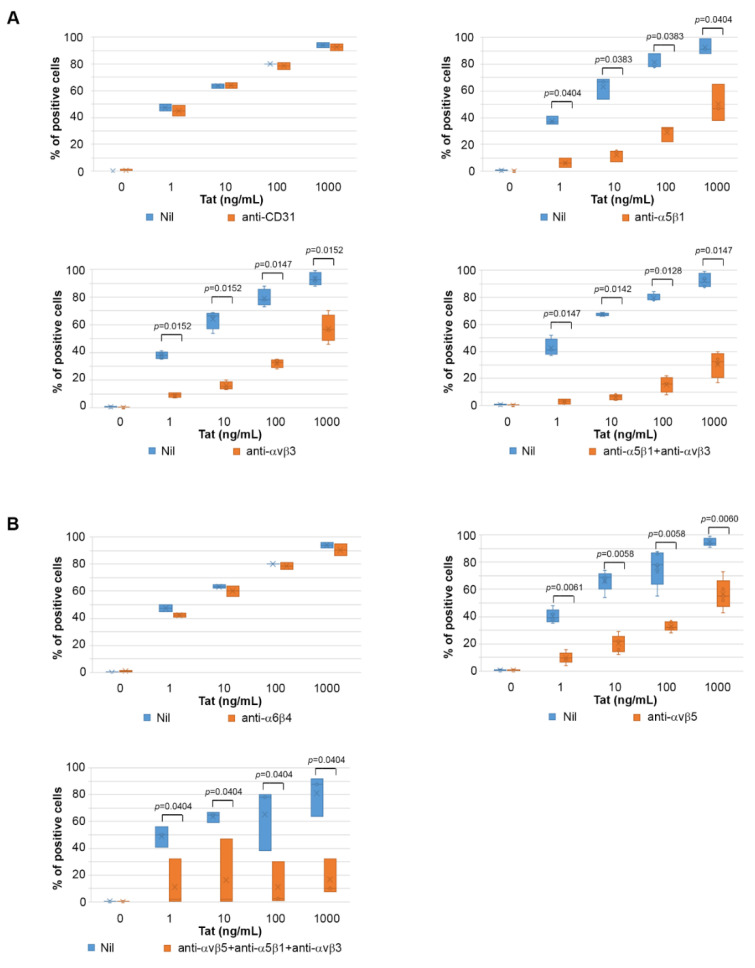
Blockade of Tat entry in IC-HUVEC by antibodies directed against the α5β1, αvβ3, and/or αvβ5 integrin. In (**A**), activated HUVEC were suspended and then incubated for 2 h with 5 μg/mL of monoclonal antibodies (mAb) directed against CD31 (upper left panel), α5β1 (upper right panel), or αvβ3 (lower left panel), or with anti-α5β1 and anti-αvβ3 mAb, combined together at 5 μg/mL each (lower right panel). In (**B**), IC-HUVEC were suspended and then incubated for 2 h with 5 μg/mL of anti-α6β4 mAb (upper left panel), or with 5 μg/mL of anti-αvβ5 mAb, alone (upper right panel) or combined with anti-α5β1 and anti-αvβ3 mAb (lower panel). For both (**A**,**B**), IC-HUVEC incubated with Ab dilution buffer (PBS-0.1% BSA) were employed as controls (Nil). After incubation, IC-HUVEC was exposed for 10 min to biologically active Tat (1–1000 ng/mL) or its buffer. Intracellular Tat content was assayed by intracellular staining and flow cytometry as described. Results are expressed as the percentage of positive cells as compared to isotype-stained samples and non-permeabilized cells. Box-plot data obtained from three to four independent experiments and analyzed by the Mann–Whitney test are shown. Dots indicate individual measures. Orange plots refer to IC-HUVEC incubated with the said mAbs; blue plots refer to control IC-HUVEC.

Results indicated that while the prior incubation of IC-HUVEC with anti-CD31 mAb had no effect on the uptake of Tat, at all Tat concentrations tested (Figure 4A), prior incubation of IC-HUVEC with anti-α5β1 or anti-αvβ3 mAbs inhibited to a comparable extent the entry of Tat, and the inhibition was again more pronounced at the low Tat concentrations (*p* = 0.0404 and *p* = 0.0152 respectively, Figure 4A). When anti-α5β1 and anti-αvβ3 mAbs were combined, a higher inhibitory effect was observed at all Tat concentrations (*p* < 0.05, Figure 4A). Thus, mAbs directed against the RGD-binding α5β1 and αvβ3 integrins effectively competed out Tat binding to IC-HUVEC and specifically abolished the entry of low Tat amounts. In substantial agreement with the results obtained with the Tat RGD peptide, the inhibition was only partial at the highest Tat concentrations, even when anti-α5β1 and anti-αvβ3 mAbs were used in combination.

To further investigate the contribution of RGD-binding integrins to Tat entry into IC-HUVEC, the role of αvβ5 was also evaluated, since this integrin has been reported to bind both the basic domain and the RGD region of cellular or microbial molecules [58,59]. Antibodies directed against the laminin receptor α6β4, which does not belong to the group of RGD-binding integrins [60], were employed as a control. Results indicated that pre-incubation with the anti-αvβ5 mAb, but not with anti-α6β4 mAb, inhibited IC-HUVEC uptake of 1, 10, 100, and 1000 ng/mL of Tat by 75, 68, 59, and 41%, respectively (*p* < 0.05 for all, Figure 4B).

Of note, when IC-HUVEC were pre-incubated with saturating amounts of mAbs directed against all the three RGD-binding integrins α5β1, αvβ3, and αvβ5, virtually no Tat uptake was observed up to the 100 ng/mL concentration of Tat, while at the highest concentration tested (1000 ng/mL), Tat entry was reduced by 89% (*p* = 0.0404, Figure 4B).

Altogether, these results indicate that α5β1, αvβ3, and αvβ5 integrin receptors mediate to a similar extent the entry of Tat in IC-HUVEC, and that the blockade of all of them is required to abolish the binding and entry of Tat at all concentrations used, which appears to be strictly dependent on the Tat RGD domain for the integrins α5β1 and αvβ3, and (conceivably) on both Tat basic and RGD domains for the integrin αvβ5.

### 2.5. The Tat RGD Domain Is Exposed and Binds αvβ3 in Modeling–Docking of Tat-Integrin Interaction

**Figure 5:**
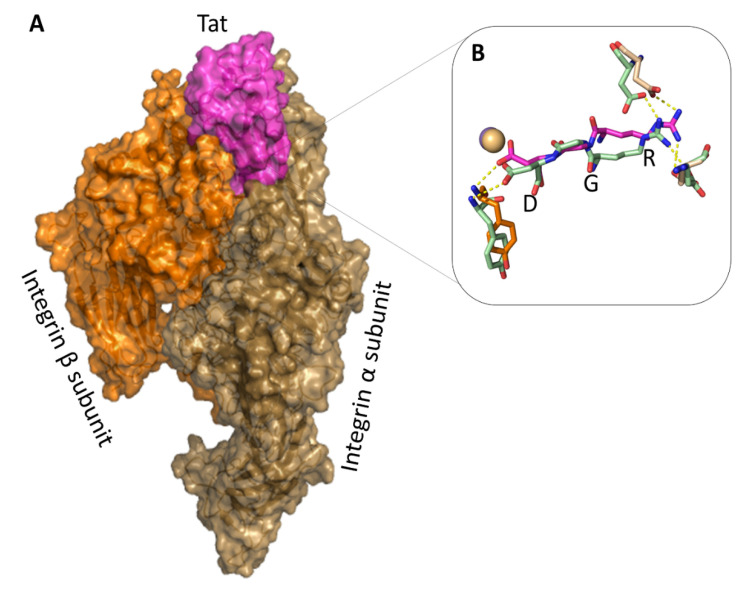
Tat makes contacts with both the α and β chains of αvβ3. (**A**) Average structural model of the Tat–αvβ3–integrin complex structure. Tat (magenta) interacts with both integrin subunits (brown = α; orange = β). (**B**) The calculated structural model of the αvβ3–Tat protein complex (magenta) is compared to the experimental structure of the αvβ3–RGD containing cyclic peptide complex (light green). Conserved hydrogen bonds between the two structures are shown. The metal ion interacting with the RGD fragment is represented as a sphere.

To gain more insights into the Tat binding to integrins, structural models of the interaction between Tat and αvβ3 were generated using the X-ray structure of the integrin αvβ3 in complex with an RGD-containing cyclic peptide [61] through docking calculations. To this purpose, three structural models were built for Tat using three experimental structures available for different strains [62]. Despite their high sequence identity, these structures showed different conformations where the RGD sequence was exposed or partially or almost completely buried. When the RGD was buried, only high-energy complexes between integrin αvβ3 and Tat were obtained, indicating that these Tat conformations are not able to induce productive binding with the integrin. Conversely, one cluster of low-energy complexes was obtained with the Tat conformation exposing the RGD sequence. Of note, in all complexes of this cluster, Tat makes contact with both the αv and the β3 subunits (Figure 5A).

In this low energy structural model, the Tat RGD region is inserted into the crevice between the β-propeller and the βA domains of the αvβ3, as observed in the X-ray structure of the complex between the RGD-containing cyclic peptide and αvβ3 (Figure 5B) [61]. Furthermore, as for the RGD cyclopeptide, Tat Asp80 interacts with the metal ion of the integrin βA domain and with Tyr1122 of the integrin β subunit, whereas Tat Arg78 interacts with both Asp150 and Asp218 of the integrin α chain (Table 1).

Additional contacts, such as hydrogen bonds and hydrophobic interactions, are present between the C-terminal region of Tat and both integrin subunits (Table 1). Furthermore, Tat Glu86 forms two salt bridges with the side chains of Arg248 and Lys1253 in the two integrin’s chains. These data suggest that the last 31 residues of Tat (56–86) containing the RGD sequence are the most important for the Tat–integrin interaction. Electrostatic complementarity of the surfaces on the two partner proteins appears to further stabilize the complex. No residue of the basic region, with the only exception of Arg56, interacts with the integrin in this model.

### 2.6. Tat Mediates Productive HIV-1 Infection of IC-HUVEC

To evaluate whether Tat entry could have an impact on endothelial cell susceptibility to HIV-1 infection, IC-activated or control HUVEC were exposed to NL4.3, a clade B X4-tropic virus, in the absence or presence of 1 µM (10 µg/mL) of biologically active Tat versus a mutated Tat protein devoid of trans-activating capability (Tat_cys22_) that was employed as control.

**Figure 6:**
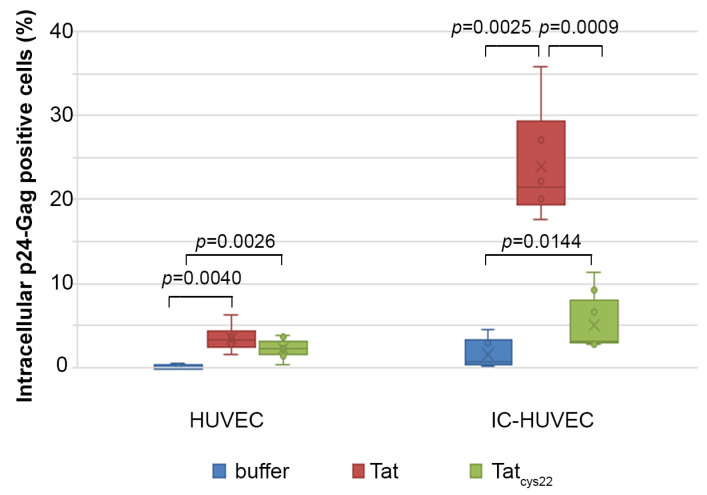
Extracellular Tat protein promotes productive Human Immunodeficiency Virus (HIV-1) infection of IC-HUVEC. HUVEC or IC-HUVEC were exposed to NL4.3 virus (p24-Gag = 70 ng/mL) in the absence or in the presence of 1 µM (10 µg/mL) of the Tat protein, either biologically active (Tat), or devoid of transactivating activity (Tat_cys22_). Four days later, cells were washed, trypsinized, and processed for intracellular staining for p24-Gag antigen. Box-plot data obtained from three independent experiments and analyzed by the Wilcoxon Two-sample Test are shown. Dots indicate individual measures.

In non-activated HUVEC, HIV-1 entry was undetectable in the absence of Tat and barely detectable in the presence of Tat (p24-Gag+ cells: 3.5%) or Tat_cys22_ (p24-Gag+ cells: 2.2%) (Figure 6). Similarly, a negligible proportion of IC-HUVEC stained positive for p24-Gag in the absence of Tat (p24-Gag+ cells: 1.5%); however, when the infection was carried out in the presence of biologically active Tat, 23.9% of the cells stained positive for intracellular p24-Gag in flow cytometry (Figure 6). This proportion was 5-fold higher than that recorded in the presence of Tat_cys22_ (p24-Gag+ cells: 5.07%) (Figure 6) despite Tat_cys22_ entering IC-HUVEC to the same extent of wild-type Tat (data not shown), as previously observed with dendritic cells [37].

**Figure 7:**
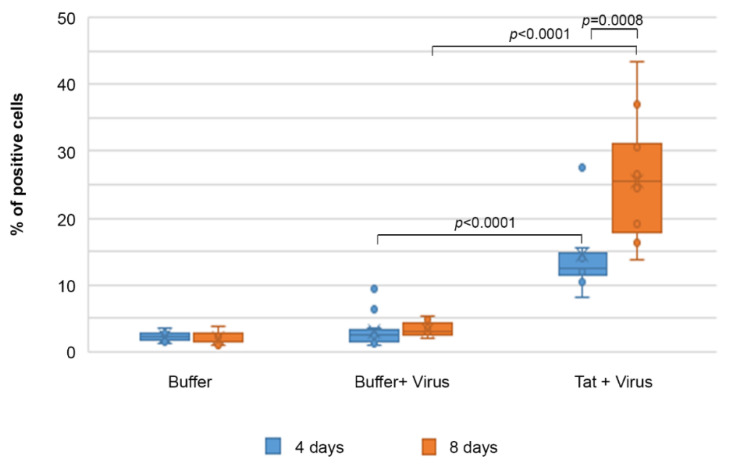
Extracellular Tat protein promotes productive HIV-1 infection of IC-HUVEC. IC-HUVEC were exposed to NL4.3 virus (p24-Gag = 70 ng/mL) in the absence or in the presence of 10 µg/mL of biologically active Tat protein (Tat). Four and 8 days later, cells were washed, trypsinized, and processed for intracellular staining for p24-Gag antigen. Box–plot of data obtained from five independent experiments and analyzed by the Wilcoxon Two-sample Test are shown. Dots indicate individual measures.

Of importance, the infection was sustained and spread to neighbor cells over time, as the proportion of infected IC-HUVEC doubled from day 4 (12%) to day 8 (25%) post-infection even after cell trypsinization and reseeding at day 4 (Figure 7).

Thus, HUVEC pre-activated with inflammatory cytokines become susceptible to HIV-1 productive infection only in the presence of biologically active Tat, indicating that it retains its biological activity upon entry and it trans-activates the incoming virus, ensuring the productive infection of endothelial cells.

### 2.7. Efavirenz Suppresses the Tat-Mediated HIV-1 Infection of IC-HUVEC

**Figure 8:**
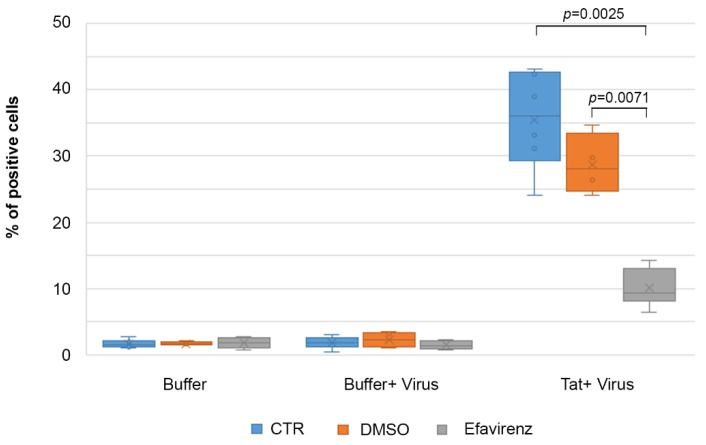
Efavirenz blocks HIV-1 productive infection of IC-HUVEC promoted by extracellular Tat protein. IC-HUVEC were pre-treated with Efavirenz (500 nM) or its resuspension buffer (0.005% DMSO) for 1 h prior to virus infection; then, they were exposed in the presence of the drug to NL4.3 virus (p24-Gag = 70 ng/mL), in the absence or in the presence of 10 µg/mL of biologically active Tat protein (Tat). Four days later, cells were washed, trypsinized, and processed for intracellular staining for p24-Gag antigen. Box-plot of data obtained from three independent experiments and analyzed by the Wilcoxon Two-sample Test are shown. Dots indicate individual measures.

To further verify whether the intracellular p24 detected into IC-HUVEC upon exposure to Tat was indeed a productive infection, experiments were carried out in the presence of the HIV-reverse transcriptase inhibitor Efavirenz. As shown in Figure 8, pretreatment with Efavirenz reduced from 35.4% to 10.1% the percentage of p24 Gag+ cells detected at day 4 in the presence of Tat protein, confirming that the main effect of Tat on IC-HUVEC is to promote productive HIV infection. Thus, Tat enters IC-HUVEC upon binding to RGD-binding integrins at pico-nanomolar concentrations and to HSPG at higher (micromolar) concentrations. Once internalized, Tat transactivates HIV gene expression, turning endothelial cells from a poorly susceptible to a relatively highly permissive phenotype and source of new virus production.

## 3. Discussion

Here, we have shown that the Tat protein of HIV is efficiently taken up by cytokine-activated endothelial cells, as previously observed with dendritic cells [37,38]. In particular, unlike the uptake occurring in the micromolar range, the entry of Tat at nano-micromolar concentrations into endothelial cells occurs only upon their activation by inflammatory cytokines, requires a biologically active protein, and it is hampered at 4 °C. These findings suggest that the entry of low amounts (1–100 ng/mL) of Tat into IC-HUVEC is a conformation- and energy-dependent process, which is likely to be mediated by specific cell surface receptors activated by inflammatory mediators. Consistently, the prior incubation of IC-HUVEC with fibronectin or vitronectin, the Tat RGD peptide, or with α5β1, αvβ3, or αvβ5 antagonists significantly reduces the entry of Tat. The blocking of both α5β1 and αvβ3 has additive inhibitory effects, and the entry of micromolar Tat concentrations (1 µg/mL) is almost completely abolished when cells are pre-incubated with a cocktail of mAbs directed against α5β1, αvβ3, and αvβ5 integrins, which is in agreement with the reported “cross-talk” occurring among these integrins. These results indicate that α5β1, αvβ3, and αvβ5 integrins are the main receptors responsible for the entry of Tat in IC-activated HUVEC, which is consistent with the ability of these integrins to mediate the cellular uptake of RGD-containing molecules [63,64,65,66,67].

IC-HUVEC take up Tat very quickly (after just 5 min). This does not occur in non-activated HUVEC. The timing of Tat entry into the IC-HUVEC is identical to that previously observed in dendritic cells [37]. In other cell types such as, for example, epithelial cells, the uptake of Tat takes longer [68]. This difference correlates with the different mechanisms underlying the two types of Tat uptake. In fact, Tat entry into epithelial cells requires high (micromolar) amounts of the protein and is mediated by the interaction between its basic residues and the HSPGs of the cell membrane [68]; in contrast, the entry of Tat into dendritic cells and cytokine-activated endothelial cells occurs at lower concentrations of the protein (1–100 ng/mL) and is mediated by RGD-binding integrins.

In this regard, it is noteworthy that inflammatory cytokine-activated endothelial cells display antigen processing and presentation capabilities, which are typical features of dendritic cells [69]. Relationships between these two cell types go further, as dendritic cells may acquire an endothelial cell phenotype under specific stimuli, [70] and monocytes may differentiate in both dendritic and endothelial cell types [71,72].

In this context, we found that inflammatory cytokines increased the expression of the αvβ5 but not the α5β1 and αvβ3 integrins on endothelial cell surface; however, inflammatory cytokines augmented endothelial cell adhesion onto fibronectin and vitronectin, which are the natural ligands of these receptors. Specifically, the ratio of IC-HUVEC to HUVEC adhesion to fibronectin coated at 0.1, 1, and 10 μg/mL was 1.67, 1.33, and 1.14, respectively. Similarly, the ratio of IC-HUVEC to HUVEC adhesion to vitronectin coated at 0.1, 1, and 10 μg/mL was 2.0, 1.5, and 1.33, respectively. Altogether, these data indicate that under our experimental conditions, inflammatory cytokines enhance the function of RGD-binding integrins.

The results of modeling–docking calculations, which have been strengthened by blocking experiments of Tat uptake with mAbs directed against the single αv chain as compared to mAbs binding the αvβ3 heterodimer, indicate that Tat and the αvβ3 integrin form a stable complex where the RGD region of Tat contacts both chains, as known for the structure of the integrin in complex with a cyclic RGD peptide. Additional extensive interactions are predicted to occur in this structure model between the C-terminal region of Tat and both integrin subunits, whereas the Tat basic sequence does not appear to interact with αvβ3. These data further support the conclusion that the basic region of Tat is not directly involved in binding to this integrin. Accordingly, Tat can concomitantly bind integrins with the RGD domain and HSPG with its basic region. However, our data indicate that Tat enters IC-HUVEC via RGD binding integrins at low (1–100 ng/mL) concentrations, whereas at higher concentrations (1 µg/mL), Tat binds HSPG and possibly enters cells via an integrin-independent pathway, as entry is only partially blocked by competition with a Tat-RGD peptide. Intriguingly, the blockade is complete when mAbs against the three RGD-binding integrins are used, suggesting some level of cooperation/interference between the two pathways at high Tat concentrations. Alternatively, differences between mAbs and Tat peptides with regard to steric hindrance and affinity for their ligands should be considered.

These findings are of relevance, as endothelial cells have been reported to be highly dysfunctional or injured in HIV infection [24,73,74,75]. This is consistent with the known role of inflammatory cytokines, which are markedly elevated in HIV-infected individuals, in inducing endothelial cell activation and dysfunction [17,18,19,20,21,22,76,77,78]. Of note, activated and dysfunctional endothelial cells become the target of Tat that, in turn, further alters their functionality. In fact, in the pre-ART era Tat has been the first HIV-1 protein reported to severely affect endothelial cells, promoting vascular endothelia dysfunction and the onset and progression of the angioproliferative KS [40,79]. More recently, a role for Tat in promoting the occurrence of cardiovascular diseases, whose prevalence is greatly increased even in successfully ART-treated individuals, has also been proposed [80]. This is consistent with the persistent inflammation and immune activation during suppressive antiretroviral therapy, which maintain endothelial cells dysfunctional and responsive to Tat that continues to be expressed despite virus suppression, as evidenced by seroconversion for anti-Tat antibodies in treated patients [80,81]. As inflammatory cytokines are strong inducers of endothelial cell activation and dysfunction, this evidence indicates a role for extracellular Tat in the pathogenesis of HIV comorbidities (AIDS-associated and non-AIDS related) that goes beyond its role in the virus life cycle and is characterized by the many ways extracellular Tat affects the physiology of the vascular system, contributing to the onset of endothelial cell dysfunction and progression toward virtually all HIV-related comorbidities.

Here, we also show that the addition of soluble, biologically active Tat to dysfunctional endothelial cells (i.e., pre-activated by TNF-α, IL-1β, and IFN-γ), and exposed to cell-free HIV-1 efficiently promotes productive HIV infection. In these experiments, different concentrations of Tat protein were used; however, the dose of Tat 10 μg/mL gave the most consistent results, as compared to lower Tat concentrations.

Several and non-mutually exclusive reasons may account for this. In particular, the intracellular availability of biologically active Tat in the infection experiments is critical, as the transactivating activity of Tat is an absolute requirement for HIV gene expression and replication. In fact, no productive infection occurs when the mutated, transactivation silent Tat_cys22_ protein is used in place of wild-type, transcriptionally active Tat. However, in our in vitro model, the intracellular levels of biologically active Tat may be reduced due to Tat stickiness to the cell membrane or extracellular matrix [39]. Furthermore, as for dendritic cells, also cytokine-activated endothelial cells rapidly digest internalized proteins [82,83,84,85]. In accordance with this, we have found that intracellular Tat levels begin to drop 30 min after entry in IC-HUVEC (Figure S1), as previously observed for dendritic cells [37].

On the other hand, the amounts of Tat (1–500 ng/mL) detected in the sera of HIV-infected individuals [51,52,53] are likely to be much lower than those present in the tissues where the virus resides. In fact, immediately after its release by acutely infected leukocytes, Tat binds the extracellular matrix [39,44], increasing leukocyte adhesiveness to endothelial cells [50], thereby placing HIV-infected, Tat-releasing leukocytes in close proximity with endothelial cells. Accordingly, Tat concentrations in solid tissues are higher than those used in our experimental model.

Although Tat can upregulate the expression of HIV co-receptors [52], no productive infection occurs when the mutated Tat_cys22_ protein (which is devoid of transactivating capacity) is used in place of wild-type, transcriptionally active Tat. In addition, kinetics of endothelial cell infection demonstrates that the percentage of p24-positive IC-HUVEC increases over time. Furthermore, the HIV-reverse transcriptase inhibitor Efavirenz strongly reduces Tat-induced HIV infection of IC-HUVEC. Altogether, these results indicate that Tat directly induces productive HIV infection in IC-HUVEC. As most of the Tat protein produced during virus replication is released extracellularly, HIV-1 infected endothelial cells may represent a new source of Tat, which may maintain and fuel HIV infection, the endothelial cell dysfunction, as well as the dysregulation of the immune system and central nervous system, even in individuals on effective cART. Of importance, the tight interactions between endothelial cells and transmigrating mononuclear cells suggest that extracellular Tat may sustain cell-to-cell HIV transmission at the time of lymphocytes and monocytes extravasation, particularly in an inflammatory environment, thus fuelling infection and/or virus reactivation in latently infected cells. Of importance, HIV-1 infected endothelial cells are hardly attackable by antiretroviral drugs, as the tight cell-to-cell contacts occurring during the trans-endothelial migration of leukocytes are likely to strongly facilitate virus transmission while limiting ART efficacy [86].

As endothelial cells are among the most represented cell type in the body, their contribution to HIV dissemination and reservoir formation and maintenance is probably very relevant. Indeed, activated monocytes and lymphocytes have been observed to accumulate in multiple perivascular foci distributed at random in the vasculature, which was reported in the pre-ART era, suggesting the potential contribution of endothelial cells to HIV dissemination in the primary infection and maintenance in the chronic phase of infection [87,88,89]. This may help explain the difficulties in achieving a functional cure and in advancing toward eradication, even in individuals treated very early after HIV acquisition.

Intriguingly, naturally occurring anti-Tat Abs have been shown to delay or block progression to disease, and those induced by Tat vaccination of individuals on effective ART appear to promote the restoration of immune homeostasis in ART-treated subjects and to exert an effective antiviral response at the portal of entry in vivo in non-human primates (reviewed in [90,91,92,93]). Taken together, these published evidence and the present findings indicate that Tat represents a major virulence factor, driving HIV replication and dissemination, inducing dysregulation of the immune, vascular, and central nervous systems, and eventually leading to the occurrence of severe comorbidities despite suppressive therapy.

## 4. Materials and Methods

### 4.1. Tat Protein Production and Purification

The HIV-1 Tat protein and the mutated, transactivation silent, Tat_cys22_ protein from human T lymphotropic virus type IIIB-BH-10 (subtype B) were expressed in *Escherichia coli*, purified to homogeneity by heparin affinity chromatography and reverse HPLC, and handled as described previously [34,37,40,57]. Three different Tat preparations were used with reproducible results. In all lots, endotoxin concentration was below the detection limit (<0.02 EU/μg), as determined by the Lymulus Amoebocyte Lysate analysis (Pyrochrome, Associates of Cape Cod, Falmouth, MA, USA). The purified Tat protein was fully biologically active, as tested by the rescue assay on HLM-1 cells carrying a Tat-defective HIV provirus [86], and by monocyte-derived dendritic cell uptake [37]. In some experiments, the recombinant Tat protein was inactivated by exposing it to air and light for 18 h at room temperature [40,94]. Oxidized Tat changed its conformation and underwent multimerization, as revealed by SDS-PAGE and analysis of its elution profile by HPLC [38,95]. Oxidized Tat was no longer capable of promoting the replication of Tat-defective proviruses, as determined by the HLM-1 assay [38,40]. Tat_cys22_ is a clade B Tat protein carrying a cysteine to glycine substitution at position 22 (Tat_cys22_), which renders the protein transactivation-silent [96], and it incapable of inducing dendritic cell maturation, although it is taken up by monocyte-derived dendritic cells as well as wild-type Tat [37,97], indicating retention of the conformational features required for binding to HSPG and/or to RGD-binding integrins and cell entry.

### 4.2. Reagents

Gelatin (denatured collagen I, from bovine skin), vitronectin (from human plasma), bovine serum albumin (BSA, fraction V) and Efavirenz were obtained from Sigma-Aldrich (Milan, Italy). Human recombinant IL-1β, TNF-α, and IFN-γ, and fibronectin from human plasma were purchased from Roche Molecular Biochemicals (Indianapolis, IN, USA). MAbs directed against the α5β1, αvβ3, αvβ5, or α6β4 integrins were obtained from Chemicon-Merck-Millipore (Darmstadt, Germany). The anti-CD31 (PECAM-1) mAb was purchased from Santa Cruz Biotechnology (Dallas, TX, USA). The Tat peptides (11–25), (46–60), and (66–80) were from UFP Service, University of Ferrara, Italy. Phosphate-buffered saline (PBS) solution, cell growth medium (RPMI 1640) and media supplements were obtained from Invitrogen-Life Technologies (Milan, Italy). Fetal bovine serum (FBS) was from HyClone (Logan, UT, USA).

### 4.3. Endothelial Cell Culture and Activation

HUVEC were obtained from Lonza (Verviers, Belgium) and grown in RPMI 1640 containing 15% FBS and supplemented with heparin, endothelial cell growth supplement, sodium pyruvate, and essential and non-essential amino acids (complete growth medium) (Invitrogen-Life Technologies, Milan, Italy). HUVEC activation was performed by culturing the cells for a total of 5 days (3 days in complete growth medium and 2 days in RPMI-15% FBS) in the presence of human recombinant IFN-γ, IL-1β, and TNF-α, combined together at concentrations (10 U/mL, 5 ng/mL, and 2 ng/mL, respectively) comparable to those found in conditioned media from activated or transformed leukocytes [40]. HUVEC activation by inflammatory cytokines was revealed by the induction of expression of cell surface ICAM-1 (CD54), as determined in flow cytometry upon staining with a fluorochrome-conjugated mAb directed against CD54 (Immunotech, Marseille, France).

### 4.4. Evaluation of Tat Protein Cellular Uptake by Flow Cytometry

Cytokine-activated or non-activated HUVEC were suspended via the use of Cell Dissociation Solution Non-enzymatic 1x (Sigma-Aldrich, Milan, Italy) and incubated for 5, 10, or 30 min at 37 °C (or at 4 °C for endocytosis-blocking experiments), in the dark, in RPMI 1640–15% FBS containing serial dilution of Tat or its suspension buffer (PBS-0.1% BSA). For blocking experiments, prior to their exposure to Tat or control buffer, cells were incubated upon continuous gentle shaking for 2 h at 4 °C with Tat peptides, fibronectin, vitronectin, or with mAb directed against α5β1, αvβ3, αvβ5, α6β4, or CD31. Incubation with the peptides, fibronectin, vitronectin, or mAb dilution buffer (PBS-0.1% BSA) was employed as a control. The mAbs were used at a final concentration of 5 µg/mL each. The Tat peptides were employed at 100 µg/mL each, while fibronectin and vitronectin were combined at 50 µg/mL each.

After their exposure to Tat or control buffer, cells were washed with cold medium and treated for 10 min at 37 °C with 0.05% trypsin to remove any externally bound protein. After fixation and permeabilization, cells were stained with affinity-purified rabbit polyclonal anti-Tat immunoglobulin G (IgG) Ab [37,97], or rabbit IgG control Ab (ICN Biomedicals, Opera, Italy), followed by fluorescein-isothiocyanate (FITC)-conjugated anti-rabbit Ig (Pierce, Rockford, IL, USA). Fluorescence was analyzed by flow cytometry, and results were expressed as the percentage of positive cells as compared with isotype-stained samples [37,97]. To demonstrate the specific intracellular localization of the protein, staining with anti-Tat Ab was always performed also with non-permeabilized cells.

### 4.5. Confocal Microscopy

To determine whether Tat enters adherent IC-HUVEC, the same short-term endocytosis pulse-chase assay previously developed to visualize the internalization of RGD-containing extracellular matrix molecules was utilized [98]. Specifically, Tat protein was labeled with rhodamine at lysine residues as described [55]. To ensure that biological activity was maintained, rhodamine-labeled Tat was tested for its capability of promoting vascular cell growth [34]. Then, IC-HUVEC were incubated at 4 °C in RPMI-15% FBS containing 100 ng/mL of rhodamine labeled-Tat (pulse phase). After 60 min of incubation, cells were washed and then incubated at 37 °C in RPMI-15% FBS in the absence of labeled Tat (chase phase). After 30 min, cells were washed, fixed with 4% paraformaldehyde, permeabilized in PBS-0.3% Triton X-100 (Sigma-Aldrich), and then observed and photographed at the confocal microscope, as described [98].

Images were taken on an inverted microscope (Olympus, Tokyo, Japan) equipped with a confocal spectral imaging system (Olympus Fluoview 1000) using a (Olympus) planapo objective 60× oil A.N. 1.42. Excitation light was obtained by a Laser Dapi 408 nm for Dapi and Diode Laser HeNe (561 nm) for Tetramethylrhodamine (TRITC). Emitted fluorescence was recorded during single excitation sessions in the same field, and same conditions for stack images collection. DAPI emission was recorded from 415 to 485 nm, TRITC emission was recorded from 568 to 678 nm. Images recorded have an optical thickness of 0.40 micrometers. Merging was obtained by freeware software “ImageJ” (vers1.29) from NIH, USA

### 4.6. Docking Calculations of the Tat-Integrin Complex

Docking calculations between integrin αvβ3 and Tat BH10 were performed with the program HADDOCK2.2 [99], where the docking process is driven by distance restraints between residues that are involved in the intermolecular interaction (http://www.nmr.chem.uu.nl/haddock). The structures of both proteins in the complex are also required as input. The input structure of integrin αvβ3 was taken from the PDB [62] (PDB identifier: 1L5G) [61]. For Tat BH10, three different input structures were used, which were generated by homology modeling with the program MODELLER9.17 [100]. The templates for the modeling were also taken from the PDB [PDB identifiers: 1JFW [101,102], and 1TBC (unpublished)]. These structures of the Tat protein are from different subtypes of the HIV-1 virus, and they were all used because despite their high sequence identity (average: 80%), they show quite different conformations (average RMSD: 10.0Ă). In particular, the RGD segment is solvent exposed (accessibility: ≈40%) in only one of the three structures, whereas in the other two, only Arg78 is partially solvent exposed, while Gly79 and Asp80 are largely buried (accessibility: ≈10%).

The distance restraints used in the HADDOCK calculations were generated as described in the HADDOCK protocol [99], by defining the residues at the interface for each of the two proteins in the complex (Table 1). The residues were chosen based on the available structure of the complex between integrin and a cyclic peptide that contains the RGD sequence [61], filtering out residues with solvent accessibility lower than 50%. Solvent accessibility was calculated with the program NACCESS [103].

The solutions of the docking calculations were clustered, using a threshold value of 1.5 Å for the pairwise backbone root-mean-square deviation (RMSD) at the interaction surface, and the resulting clusters were ranked according to their average interaction energy (defined as the sum of van der Waals, electrostatic energy terms) and buried surface area.

### 4.7. Infection Experiments

For the infection experiments NL4.3, an X4-tropic HIV clade B lab strain grown in Jurkat cell line was used. On day 0, IC-HUVEC or control HUVEC (1 × 10^5^/mL, resuspended in RPMI 1640 15% FBS and basic fibroblast growth factor at 10 ng/mL) were dispensed in 6-wells plates (COSTAR), pre-coated with gelatin (1.5%), and incubated at 37 °C, 5% CO_2_, 90% humidity. The following day, the RPMI 1640 15% FBS was replaced and combined IL-1β, TNFα, and IFNγ were added to the cells. The next day, IC-HUVEC were exposed contemporarily to NL4.3, (p24-Gag = 70 ng/mL), ±Tat protein (1 µM, 10 µg/mL), in a final volume of 1.2 mL. Four days later, cells were washed, trypsinized to detach them and to remove both the Tat protein and the virions bound to the cell membrane, permeabilized, and stained for intracellular p24-Gag protein (see below). In some experiments, a proportion of the detached IC-HUVEC was reseeded and cultured for an additional 4 days in RPMI-15% FBS. After that, cells underwent the same procedure to evaluate infection at day 8 post-infection. In blocking experiments, IC-HUVECs were pretreated for 1 h at 37 °C with Efavirenz (500 nM) or its resuspension buffer (DMSO, 0.005%) prior to virus exposure, and analyzed 4 days post-infection for intracellular p24-Gag protein.

For intracellular p24-Gag staining, cells were transferred to V-bottomed tubes and washed once in PBS containing 2.5% FCS. Then, cells were fixed and permeabilized using the Cytofix/Cytoperm Kit (BD-PharMingen, San Diego, CA, USA). Permeabilized cells were washed using the wash buffer provided by the manufacturer and resuspended for 60 min at 4 °C with 50 μL of a 1:50 dilution of a phycoerythrin (PE)-conjugated mouse anti-p24-Gag mAb (KC57-RD1; Beckman Coulter, Inc., Fullerton, CA, USA) or a mouse IgG1 isotypic control antibody. After two additional washes, cells were analyzed with a FACSCanto flow cytometer (Becton Dickinson), and data analysis was performed with FACSDiva software (Becton Dickinson).

Live cells initially gated by forward and side scatter were analyzed for the intracellular expression of p24-Gag. The number of p24-Gag-positive cells was determined using a bivariate plot of fluorescence versus side scatter, the gate was set on mock-p24-Gag positive cells.

### 4.8. Statistical Analysis

A Wilcoxon Two-sample Test was used. Statistical analyses were carried out at one-sided with a 0.05 significance level, using SAS^®^ (Version 9.4, SAS Institute Inc., Cary, NC, USA).

## Figures and Tables

**Table 1:** Intermolecular H-bonds conserved in at least four of the ten lowest-energy models for the Tat–integrin complex structures.

Tat-Interacting Residues	Integrin-Interacting Residues	Interaction Type
17-GLN	1126-ASP	SC-SC H-bond
56-ARG	1180-MET	MC-SC H-bond
62-SER	1214-ARG	SC-SC H-bond
75-SER	178-TYR	SC-SC H-bond
**78-ARG**	**150-ASP**	**SC-SC H-bond**
78-ARG *	218-ASP *	**SC-SC H-bond**
**80-ASP**	**1122-TYR**	**SC-MC H-bond**
**80-ASP**	**4001-MN2+**	**SC-Mt bond**
80-ASP	1123-SER	SC-MC H-bond
86-GLU	248-ARG	SC-SC H-bond
86-GLU	1253-LYS	SC-SC H-bond

* Bidentate interactions. Experimentally reported interactions [61] between the RGD domain of Tat and the integrin are shown in bold; Residues of the α_v_ integrin chain are numbered from 1 to 956, residues of the β_3_ integrin chain are numbered from 1055 to 1690; SC = side chain; MC = main chain.

## Data Availability

The data presented in this study are available on request from the corresponding author.

## Supplementary Materials

The following are available online at https://www.mdpi.com/1422-0067/22/1/317/s1, Figure S1: Extracellular Tat protein uptake by activated endothelial cells after 30 min exposure. HUVEC (blue plots) or IC-HUVEC (red plots) were incubated for 30 min in medium containing serial concentrations (1-1000 ng/mL) of biologically active Tat or its suspension buffer (PBS-0.1% BSA). The intracellular Tat content was evaluated by flow cytometry after staining with affinity-purified rabbit anti-Tat polyclonal Ab (orisotype control), as described in Section 4 Materials and Methods. Non-permeabilized cells were also analyzed by intracellular staining and flow cytometry, with negative results. Results are expressed as the percentage of positive cells, as compared to isotype-stained samples. Box–plot of data obtained from three independent experiments and analyzed by the Mann-Whitney nonparametric test are shown. Figure S2: Extracellular Tat protein is efficiently taken up by activated endothelial cells. Images of serial sections of the cell analyzed in Figure 1B,C showing the presence of Tat (red) in the optical sections of the central region of the cell.

## Abbreviations

HIV-1	Human Immunodeficiency Virus 1
HUVEC	human umbilical vein endothelial cells
RGD	arginine-glycine-aspartic acid domain
cART	combination antiretroviral therapy
HSPG	heparan–sulfate proteoglycans
IL-1	interleukin 1
TNF	tumor necrosis factor
IFN	interferon
ICAM-1	intercellular adhesion molecule-1
IC-HUVEC	inflammatory cytokines activated HUVEC
DMSO	dimethyl sulfoxide
PBS	phosphate-buffered saline
