# Supplementary material for: HIV-1 Tat Protein Enters Dysfunctional Endothelial Cells via Integrins and Renders Them Permissive to Virus Replication
Source: Int J Mol Sci. 2020 Dec 30;22(1):317. doi: 10.3390/ijms22010317 (PMC7796023; doi:10.3390/ijms22010317)
Supplement: Supplementary file 1 [file ijms-22-00317-s001.pdf]

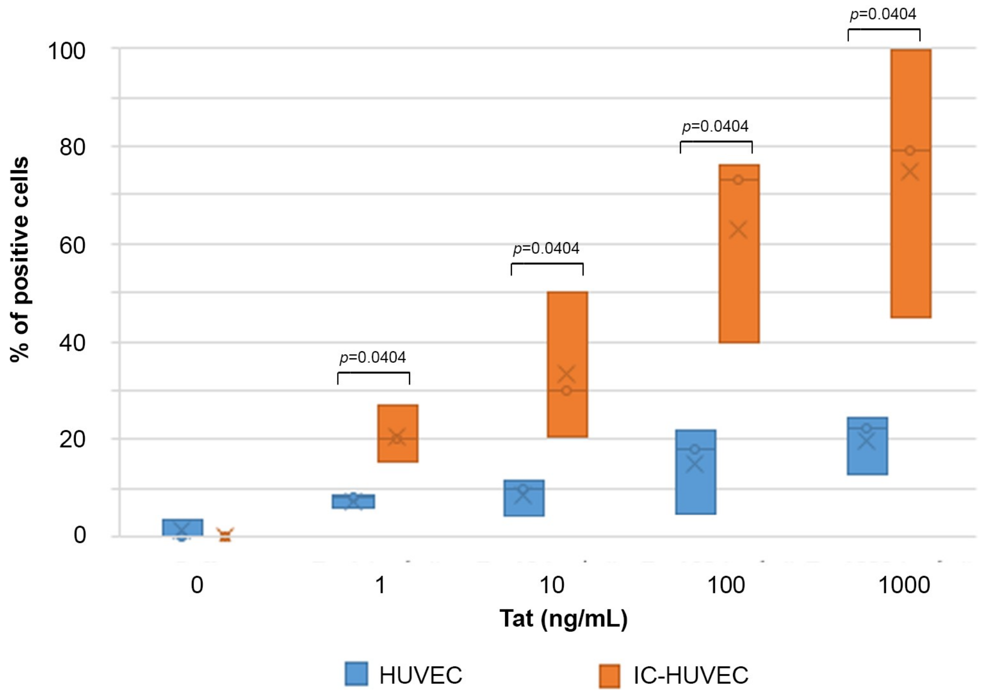

Supplementary Figure S1. Extracellular Tat protein uptake by activated endothelial cells after 30 minutes exposure. *HUVEC (blue plots) or IC-HUVEC (red plots) were incubated for 30 min in medium containing serial concentrations (1-1000 ng/mL) of biologically active Tat or its suspension buffer (PBS-0.1% BSA). The intracellular Tat content was evaluated by flow cytometry after staining with affinity-purified rabbit anti-Tat polyclonal Ab (or isotype control), as described in the Materials and Methods section. Non-permeabilized cells were also analyzed by intracellular staining and flow cytometry, with negative results. Results are expressed as the percentage of positive cells, as compared to isotype-stained samples. Box–plot of data obtained from three independent experiments and analyzed by the Mann-Whitney nonparametric test are shown.*

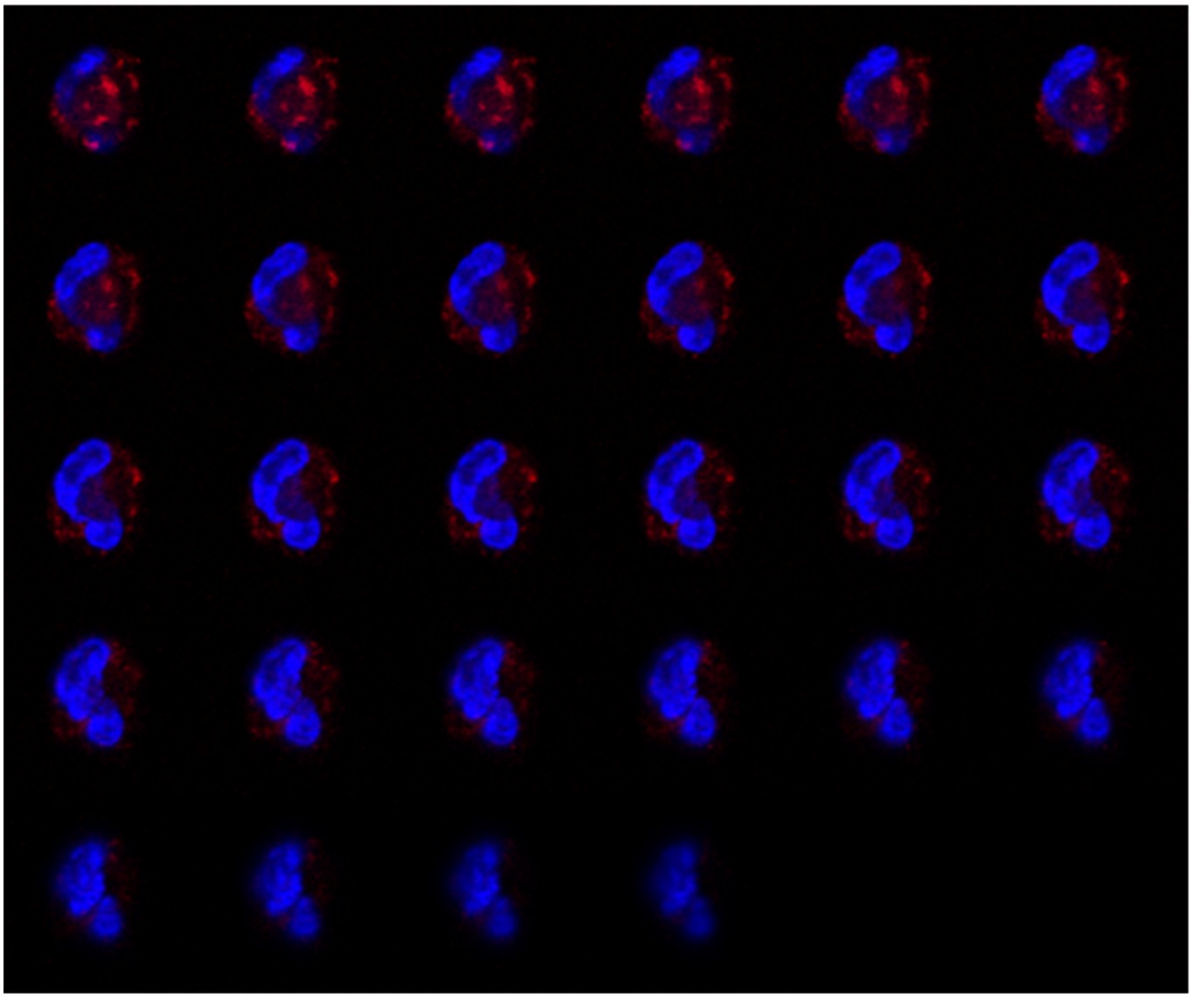

Supplementary Figure S2. Extracellular Tat protein is efficiently taken up by activated endothelial cells.
Images of serial sections of the cell analyzed in Fig. 1 B and C showing the presence of Tat (red) in the optical sections of the central region of the cell.
